# Proteomic analyses of brain tumor cell lines amidst the unfolded protein response

**DOI:** 10.18632/oncotarget.10032

**Published:** 2016-06-14

**Authors:** Jasmina S. Redzic, Joe D. Gomez, Justin E. Hellwinkel, Thomas J. Anchordoquy, Michael W. Graner

**Affiliations:** ^1^ Skaggs School of Pharmacy and Pharmaceutical Sciences, University of Colorado Denver Anschutz Medical Campus, Aurora, CO, USA; ^2^ Department of Neurosurgery, University of Colorado Denver Anschutz Medical Campus, Aurora, CO, USA

**Keywords:** brain tumor, glioma, proteomics, unfolded protein response, signaling

## Abstract

Brain tumors such as high grade gliomas are among the deadliest forms of human cancers. The tumor environment is subject to a number of cellular stressors such as hypoxia and glucose deprivation. The persistence of the stressors activates the unfolded proteins response (UPR) and results in global alterations in transcriptional and translational activity of the cell. Although the UPR is known to effect tumorigenesis in some epithelial cancers, relatively little is known about the role of the UPR in brain tumors. Here, we evaluated the changes at the molecular level under homeostatic and stress conditions in two glioma cell lines of differing tumor grade. Using mass spectrometry analysis, we identified proteins unique to each condition (unstressed/stressed) and within each cell line (U87MG and UPN933). Comparing the two, we find differences between both the conditions and cell lines indicating a unique profile for each. Finally, we used our proteomic data to identify the predominant pathways within these cells under unstressed and stressed conditions. Numerous predominant pathways are the same in both cell lines, but there are differences in biological and molecular classifications of the identified proteins, including signaling mechanisms, following UPR induction; we see that relatively minimal proteomic alterations can lead to signaling changes that ultimately promote cell survival.

## INTRODUCTION

Gliomas are the most common form of primary brain tumors and are among the deadliest of human neoplasms [1]. Among gliomas, glioblastoma multiforme (GBM) is the most aggressive and the deadliest tumor type, classified as WHO grade IV. The current standard of therapy for these tumor types is maximal surgical tumor resection, chemotherapy and radiation or a combined therapy of the aforementioned treatments [2]. Even with the use of optimal surgical interventions and therapeutic agents, survival rates are abysmally low for GBM patients, with median survival of less than 15 months.

The World Health Organization (WHO) classifies brain tumors as grade I- grade IV. These classifications are based on several factors and include genetic analysis of the tumor, histopathology, imaging results, and associated malignancy grade [3]. Oligodendrogliomas are classified as lower malignancy grade II tumors, but in ~70% of patients lead to more aggressive grade III and grade IV tumors such as anaplastic oligodendrogliomas [1, 4]. The Central Brain Tumor Registry of the United States statistical report evaluating different brain tumor types and survival rates for patients in the US between 2008-2012 showed that the 5-year survival rates are 79.8%, 52.5% and 5.1% overall for oligodendrogliomas, anaplastic oligodendrogliomas and GBMs, respectively [5], and those numbers decrease with increasing age of the patient (e.g., GBM patients >75 years of age have 0% 5-year survival rates). Despite constant efforts toward a better understanding of brain tumor biology, there has been little improvement in increasing patient survival for GBM patients in the past 20 years. It is clear that novel therapeutic targets and therapeutic interventions are critically necessary to improve outcomes for this deadly disease, and a better understanding of the tumors’ intrinsic biologies may aid in improved treatments.

Cellular stressors such as hypoxia [6], glucose deprivation [7], oxidative stress and reactive oxygen species (ROS) [8], and changes in intracellular calcium levels [9] may lead to an accumulation of unfolded or misfolded proteins. If this accumulation of unfolded proteins occurs in the endoplasmic reticulum (ER), it will lead to ER stress, which may activate the unfolded protein response (UPR). Activation of the UPR leads to an increase in the protein folding capacity of the ER by chaperone protein upregulation, an overall reduction in global protein translation (thus decreasing overall protein input), and an increase in degradation of misfolded proteins until homeostatic conditions are restored, or the cell commits to apoptosis [10, 11]. In response to the activation of the UPR, three arms of the UPR transduction pathways modulate transcriptional and translational changes necessary to return the cell to homeostatic conditions, or to instigate apoptosis [10]. Transcriptional changes are mediated by the inositol-requiring enzyme-1 (IRE1) and activating transcription factor-6 (ATF6), while translational changes are carried out by the protein-kinase RNA-like endoplasmic reticulum kinase (PERK). These ER sensors can be activated upon release from ER chaperones such as GRP78 (BiP/HSPA5) or, in some cases, with direct activation by unfolded proteins, leading to signaling cascade induction [12].

The tumor microenvironment is subject to a variety of cellular stressors such as hypoxia, glucose deprivation, and oxidative damage due to high metabolic activity of tumor cells and constant demand for macromolecules required for expansion and progression of the tumor [13]. These cellular stressors lead to the activation of the UPR and have been shown to play roles in different aspects of tumor development and progression [14]. Our lab previously showed that activation of UPR results in increased metabolism and chemo-resistance in xenograft tumors and parent cell lines [15]; however, little else is known about the role of the UPR in brain tumors [11, 16].

To gain a better understanding of the changes induced by the UPR, we assessed differences at the molecular level by global proteome analysis of cells under both homeostatic and stress conditions. Specifically, we analyzed the global proteomes of two brain tumor cell lines, U87MG, a historically well-characterized GBM (WHO grade IV) cell line, and UPN933, a primary anaplastic oligodendroglioma (AO, WHO grade III) cell line, under homeostatic (unstressed) and UPR-induced conditions (stressed). We suspected that there would be specific and/or numerical changes in the proteome outputs of the cell lines under stress that would reflect functional outcomes, including changes in signaling pathways responsive to cell proliferation or cell death. Our data show that the changes in the global proteomes between the unstressed and stressed cells are limited to about 20% of the proteome. Gene ontology (GO) analyses, pathway/network identification, and signaling alterations suggest that there are meaningful differences within and between the cell lines following UPR induction. We find that the differences are observed in the subsets of proteins unique to unstressed and stressed conditions that warrant greater exploration. Further, we find that changes induced by introduction of stress have different effects on the two cell lines studied here. Our data imply that UPR stress does not invoke dramatic proteomic changes to glioma cells, but nonetheless alters key signaling pathways while preventing apoptosis. This work stages future studies for exploring roles of specific proteins and/or groups of proteins to expand our understanding of the effects of stress conditions in the tumorigenicity of gliomas. The ultimate goal would be to utilize this information to design better treatment strategies, or at least to better understand treatment failures, from the perspective of stressed cells and their defense mechanisms.

## RESULTS

### Induction of the UPR in UPN933 cells

The UPR is a cellular mechanism that is activated under conditions of stress, leading to changes in cellular transcriptional/translational activity. Hallmarks include upregulation of ER chaperones, and activated UPR transducers and transponders. We induced the UPR in cells with the reducing agent dithiothreitol (DTT), which disrupts disulfide bonding in the oxidizing environment of the ER and is widely used and validated for that purpose [17]. We previously demonstrated UPR induction in U87MG cells [15]; in Supplementary Figure S1 we show similar Western blot results for UPN933 cells. There is noticeable upregulation of chaperones GRP170, calnexin, ERp72, calreticulin, and HERPUD. The UPR transducer ATF6 shows increased amounts of the active transcription factor forms (p60, p36) with a concurrent reduction in the full-length p90 form. Similarly, there is more XBP1 in the active (spliced) form with a reduction in the unspliced form; the transcription factor CHOP/GADD153 is also upregulated. These results indicate successful UPR induction in the UPN933 cells. We should note that the upregulation of CHOP/GADD153 is not necessarily a harbinger of apoptosis, as expression levels were quite variable in our previous study [15], and its subcellular localization (eg, cytosolic vs nuclear) may play a role in its biologic activity [11].

### Protein identifications via mass spectrometry

**Figure 1:**
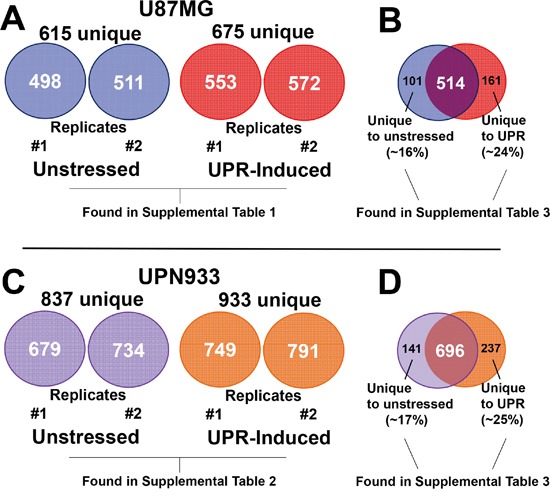
Total protein identifications Protein identifications were performed using the Mascot server searching all human entries in the SwissProt database. **A.** Number of total proteins identified in replicate samples for unstressed and stressed condition for the U87MG cells. **B.** Replicate samples were combined into a single list for comparison across conditions and cell lines. Comparison of unstressed and stressed total unique protein identifications is shown for U87MG cells. **C.** Number of total proteins identified in replicate samples for unstressed and stressed condition for the UPN933 cells. **D.** Comparison of unstressed and stressed total unique protein identifications of the combined replicate samples is shown for UPN933 cells. Also denoted are the tabulated locations for the particular datasets identified.

We used mass spectrometry analyses to identify global proteome changes in two high grade glioma cell lines under homeostatic and stress conditions. Unstressed and UPR-stressed cells were harvested and lysed; proteins were extracted from lysates from the two cell lines and conditions, and were identified via mass spectrometry techniques. Using replicate samples, we identified 498 and 511 proteins in U87MG unstressed cells, and 553 and 572 proteins in U87MG DTT-treated (UPR-induced) cells. In total, we identified 615 unique proteins from U87MG unstressed cells, and 675 unique proteins were identified from U87MG UPR-induced cells (Figure 1A). Within the combined replicate U87MG dataset shown on the right side (Figure 1B), 101 proteins (~16%) are unique to the unstressed condition, and 161 proteins (~24%) are unique to the stressed condition, with an overlap of 514 proteins common to both.

For replicates of the UPN933 cells and conditions, we identified 679 and 734 proteins in UPN933 control cells (totaling 837 proteins), and 749 and 791 (totaling 933 proteins) in UPN933 UPR-induced cells (Figure 1C). For UPN933 cells, 141 proteins (~17%) are unique to the unstressed condition, and 237 proteins (~25%) are unique to the stressed condition, with an overlap of 696 proteins common to both conditions (Figure 1D).

Protein identifications for each sample, within each condition, are presented in Supplementary Tables S1 (U87MG, unstressed/stressed for each replicate), and S2 (UPN933, unstressed/stressed for each replicate), respectively. Lists of proteins unique to each condition (only in unstressed or only in stressed for U87MG and UPN933) are presented in Supplementary Table S3.

Regarding reproducibility, we have performed additional proteomic studies on the same cell lines (J Redzic, manuscript in preparation), showing >85% overlap (for UPN933, data not shown) to over 95% overlap (U87MG) in identified proteins from unstressed/stressed conditions (Supplementary Figures S2, S3). Bioinformatic comparisons of these multiple replicates indicate a high degree of similarity despite what is a very complicated cellular process when the UPR is invoked (Supplementary Figure S4).

Replicate samples employed for this study were combined for comparison of all proteins identified in unstressed and UPR conditions. The data presented henceforth are those of the combined replicate samples.

### Gene ontology analysis using Panther database

**Figure 2:**
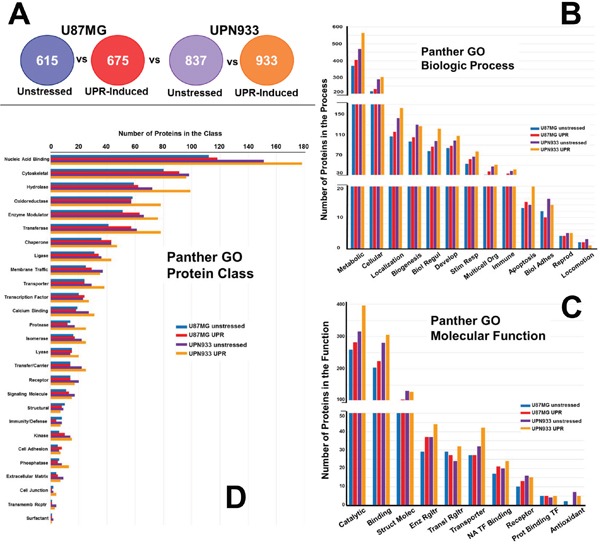
Panther database comparisons of identified proteins from entire cellular proteomes of unstressed versus stressed (UPR-induced) U87MG and UPN933 cells **A.** The sets of proteins utilized are shown. Distribution of identified proteins based on gene ontology (GO) classification of **B.** biological process; **C.** molecular function; **D.** protein class. Shown are the total numbers of proteins classified within each category for each cell line under unstressed and stressed condition. Biol Regul = Biologic Regulation; Develop = Development; Stim Resp = Response to Stimulus; Multicell Org = Multicellular Organism; Biol Adhes = Biologic Adhesion; Reprod = Reproduction; Struct Molec = Structural Molecule; Enz Rgltr = Enzyme Regulator; Transl = Translational; NA = Nucleic Acid; TF = Transcription Factor; Prot = Protein; Transmemb Rcptr = Transmembrane Receptor.

**Figure 3:**
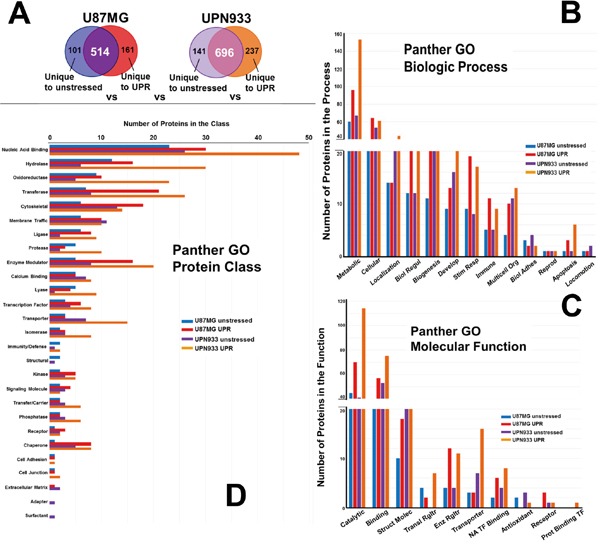
Panther database comparisons of identified proteins from proteomes unique to unstressed versus stressed (UPR-induced) U87MG and UPN933 cells **A.** The sets of proteins utilized are shown. Distribution of identified proteins based on gene ontology (GO) classification of **B.** biological process; **C.** molecular function; **D.** protein class. Shown are the total numbers of proteins classified within each category for each cell line under unstressed and stressed condition. Biol Regul = Biologic Regulation; Develop = Development; Stim Resp = Response to Stimulus; Multicell Org = Multicellular Organism; Biol Adhes = Biologic Adhesion; Reprod = Reproduction; Struct Molec = Structural Molecule; Enz Rgltr = Enzyme Regulator; Transl = Translational; NA = Nucleic Acid; TF = Transcription Factor; Prot = Protein.

**Figure 4:**
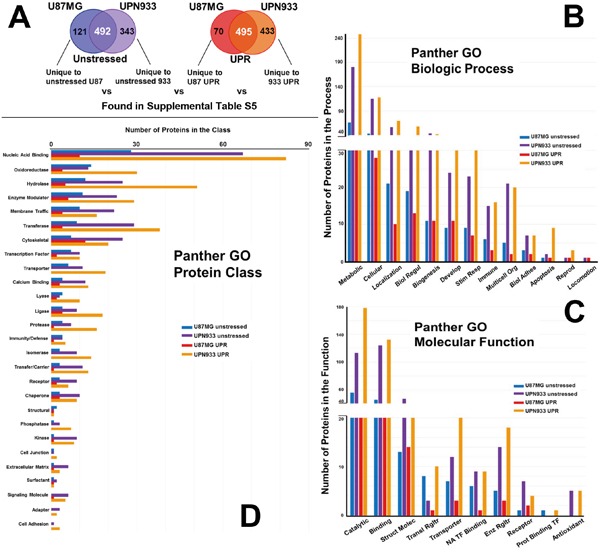
Panther database comparisons of identified proteins from proteomes unique to unstressed conditions for U87MG versus UPN933 cells, and from proteomes unique to stressed (UPR-induced) U87MG versus UPN933 cells **A.** The sets of proteins utilized are shown. Distribution of identified proteins based on gene ontology (GO) classification of **B.** biological process; **C.** molecular function; **D.** protein class. Shown are the total numbers of proteins classified within each category for each cell line under unstressed and stressed condition. Note the changes in order (bar color) of the cell lines and states. Biol Regul = Biologic Regulation; Develop = Development; Stim Resp = Response to Stimulus; Multicell Org = Multicellular Organism; Biol Adhes = Biologic Adhesion; Reprod = Reproduction; Struct Molec = Structural Molecule; Enz Rgltr = Enzyme Regulator; Transl = Translational; NA = Nucleic Acid; TF = Transcription Factor; Prot = Protein.

Panther database was used to generate gene ontology (GO) profiles based on biological process, molecular function, and protein class using our proteomic lists from mass spectrometry analyses [18]. The analyses were performed for combined replicate datasets, i.e., all of the proteins for a given condition for each cell line (Figure 2). We further compared classifications for proteins that are unique to each condition within each cell line, i.e. comparison of proteins unique for unstressed versus stressed condition for U87MG and for UPN933 cells (Figure 3). Finally, we also compared proteins that are unique to each cell line for a given condition, i.e. comparison of proteins unique to the unstressed condition for U87MG with proteins unique to the unstressed condition for UPN933 cells, as well as in their stressed conditions (Figure 4). The classification systems may provide insight into unique (or commonly-held) features of the two states and the two cell lines in the context of a defined GO classification structure. Data are presented below for the three GO classifications used.

### GO classifications comparing unstressed to stressed total cellular proteomes

#### Biologic processes

Figure 2A shows the protein sets used for GO comparisons in the following analyses (i.e., total proteins unique to unstressed and UPR-stressed conditions, for both the U87MG and UPN933 cell lines). As can be seen in Figure 2B, the distribution of proteins classified within the different biological process subsets for the whole dataset is quite similar when comparing unstressed and stressed cells for either the higher grade (grade IV, GBM) U87MG cell line or the lower grade (grade III, AO) UPN933 cells, with general increases in identified process protein members in the stressed state. This does appear exacerbated in apoptotic processes for the UPR-induced UPN933 cells. One exception is biologic adhesion, where the UPR reduces proteins in that process (and perhaps for the UPN933 cells regarding locomotion, although the numbers are small). This may imply a testable implication that the UPR either contributes to cell migration (due to reduction in adhesion) or to cellular immobility (loss of locomotion), as if the cells focus on overcoming the immediate stress threats. Generally, activation of the UPR is associated with enhanced migration/metastasis in gliomas [19, 20], but this has not been extensively studied. Overall, using the combined replicate data, we do not find major differences in the categories between the two conditions or between the cell types.

#### Molecular function

Considering the total proteome of the two cells lines under unstressed and stressed conditions, there are generally more proteins classified within these categories in the cells subjected to the stressed conditions (~10-25%, Figure 2C). However, this observed difference is likely accounted for by the change in total protein identifications, i.e., ~15-25% more proteins were identified in the stressed condition than unstressed. Nonetheless, this phenomenon is interesting in that the UPR is generally associated with attenuated protein expression [12], but fits with our previous work with U87 cells [15]. Although the trend is similar for most of the categories, we find opposing changes in total protein number for two categories. In the U87MG cells, we see a slight increase in proteins classified in the structural and receptor categories (UPR stress), but with corresponding decreases in the UPN933 cells in the stressed condition. The opposite is true for the translational regulator and transporter categories. For both cell lines, UPR stress decreases antioxidant proteins. While this may reflect a depletion of such proteins during ER stress, there may be compensatory protein activity in the ER or even cytosol [21]. Indeed, we identified a number of glutathione-related or interacting proteins in our work (Supplementary Tables S1 and S2).

#### Protein class

Protein identifications were categorized according to protein class (Figure 2D). The trend of increased representation under stress conditions occurs in one-third of the categories, with the largest subset of proteins being nucleic acid binders. This is curious in the context of ribonuclear binding proteins as components of stress granules that might be engaged during the UPR [22]. Of the more prominent categories, there are fewer cytoskeletal proteins in the stressed UPN933 cells, and fewer oxidoreductases in the stressed U87MG cells. In terms of UPR effects, chaperones and membrane traffickers are also notable, as one would expect during the UPR, along with transcription factors. The trends are not identical between the cell lines and the unstressed/stressed conditions (e.g., transporter, calcium binding, protease, lyase, signaling molecule, phosphatase, extracellular matrix, and cell junction categories) suggesting some specificity for a given cell line.

### GO classifications comparing proteins unique to unstressed/stressed conditions within a given cell line

#### Biologic process

Figure 3A depicts the origin of the protein sets for these particular studies, i.e., those proteins unique to either the unstressed state or the UPR within the U87MG or UPN933 cell lines. While the biologic process categories remain the same (Figure 3B compared to Figure 2B), and the top 3 processes (metabolic, cellular, and localization) still account for the most proteins, the rank order based on numbers of proteins differs somewhat. The trends in terms of increases or decreases following stress also remain the same, although the relative quantities display cell line dependence. Comparison of proteins unique to either unstressed or UPR conditions for U87MG and UPN933 cells show differences in the number of proteins belonging to a particular group. Of interest are changes such as those in U87MG cells for biological regulation, biogenesis, response to stimulus, multicellular organismal, and immune system processes following stress, where the number of proteins is 2-3x higher than in the unstressed group. A notable group in the “response to stimulus” category includes several chaperones/heat shock proteins or interacting proteins, again indicative of stress responses. For UPN933 cells, these larger changes in protein numbers are observed in categories of biological regulation, response to stimulus, and immune system, reflective in increases in MHC I and MHC II molecules. Both cell lines show relatively large increases in apoptotic process components following UPR induction, as might be expected.

#### Molecular function

The molecular function categories (Figure 3C) also remain the same as in Figure 2C, but again, the rank order differs for translational and enzyme regulators, antioxidants, receptors, and protein binding transcription factors. Catalytic, binding, and structural functions remain the categories with the most proteins, but the percentage differences between unstressed and stressed sets are skewed dramatically towards stress when the proteins unique to unstressed or stressed states are analyzed separately (also true of the enzyme regulator and nucleic acid transcription factor binding categories). There are similar trends between Figure 2C and Figure 3C for the rest of the categories, but again with larger protein quantity differentials between stressed and unstressed states, particularly for translational regulator and transporter categories. Exceptions are the receptor category (reduced numbers for UPN933 in both states) and the protein binding transcription factor category which have quite low protein constituents.

#### Protein class

Comparing protein classes for proteins unique to unstressed or stressed states within the cell lines (Figure 3D), and compared to Figure 2D, we note the loss of the transmembrane receptor category and replacement with the adapter category (but these both have low numbers of protein identification). With the exception of the top-ranked category (nucleic acid binding), the rank orders are considerably different. In almost all categories where the database provides 5 or more proteins, the unstressed vs stressed differentials are also more dramatic, particularly in the cases of UPR-stressed UPN933 cells (e.g., nucleic acid binding, hydrolase, oxidoreductase, transferase, protease, enzyme modulator, lyase, transporter, isomerase, transfer/carrier, and phosphatase categories). Notable in these data are the higher number of chaperone proteins in the stressed condition, as would be expected. Further, we find that the number of transcription factor proteins is also higher in the stressed groups—including molecules related to stress signaling such as PDZ and LIM domain protein 1 (PDLIM1), gamma-interferon-inducible protein 16 (IFI16), protein kinase C delta-binding protein (PRKCDBP) (for U87), and signal transducer and activator of transcription 1-alpha/beta (STAT1), hematopoietic lineage cell-specific protein (HCLS1), myb-binding protein 1A (MYBBP1A), and TAR DNA-binding protein 43 (TARDBP) (for UPN933). This suggests specificity at the molecular level upon UPR induction for these two cell lines.

A Panther database-assigned list of classifications for particular biological process, molecular function or protein class for the entire dataset is in Supplementary Table S4.

### GO classifications comparing proteins unique to unstressed/stressed conditions across the cell lines

#### Biologic process

Figure 4A depicts the origin of the protein sets for these particular studies, i.e., those proteins unique to either the unstressed state or the UPR-induced state comparing U87MG to UPN933 cell lines (protein ID lists are in Supplementary Table S5). The biologic process categories (Figure 4B) and their order are the same as for Figure 3B. The total numbers of proteins are much higher from the UPN933 cell line (~3x for the unstressed states to ~6x for the UPR states), and the differences in the protein numbers in the biologic process categories between the cell lines reflect that, with even greater differences under the stressed states. This includes proteins from UPN933 stressed cells with relationships to extracellular vesicles such as guanine nucleotide-binding protein G(s) subunit alpha isoforms short (GNAS1), double-stranded RNA-binding protein Staufen homolog 1 (STAU1), calpain small subunit 1 (CAPNS1), collagen alpha-2(VI) chain (COL6A2), transforming growth factor-beta-induced protein ig-h3 (TGFBI), serine dehydratase-like (SDSL), EH domain-containing protein 4 (EHD4), transgelin-2 (TAGLN2), and nucleolysin TIAR (TIAL1) of the “multicellular organismal” category. Varying cellular stresses result in the enhanced release of exosomes/extracellular vesicles [23, 24] (Graner et al, unpublished), further suggesting the relevance of these proteins. The ratios between the UPR-induced UPN933 cells and the U87MG cells are especially notable for the cellular, localization, response to stimulus, immune, multicellular organismal, and apoptosis categories.

#### Molecular function

Figure 4C compares the numbers of proteins between U87MG and UPN933 lines unique to their unstressed or stressed states in molecular function categories. These categories are the same as in Figure 3C, but beyond the high-ranking catalytic, binding, and structural categories, their rank orders differ from both previous graphs. The same trends of increased proteins in the UPR state for UPN933 are again evident, with high differentials in the catalytic, binding, translational regulator (for UPN933, numerous members of the 43S-pre-initiation complex [25]), transporter, and nucleic acid transcription factor binding categories. We again do not identify any proteins belonging to the antioxidant category in the U87MG stressed cells. For the unstressed states, U87MG has more proteins in the translational regulator and protein binding transcription factor categories than UPN933, going against the general trend.

#### Protein class

Comparing protein classes for proteins unique to unstressed or stressed states between the cell lines (Figure 4D), and compared to Figure 2D and 3D, the classes are the same as Figure 3D, but again with the exception of the nucleic acid binding class, are often in a different rank order. For the nucleic acid binding class, stressed U87 cells express numerous splicing factors, while the stressed UPN933 cells produce many ribosomal proteins and translational initiation factors/subunits. The general trend continues to reflect the abundance of proteins from UPN933 (both unstressed and UPR state) over U87MG, particularly noticeable, and perhaps over-represented, in the hydrolase, transferase, transporter, protease, isomerase, transfer/carrier, phosphatase, kinase, cell junction, signaling molecule, adapter, and cell adhesion categories (where 6 of those are void in the U87MG UPR state).

A general theme from these studies, particularly those comparing unstressed vs stressed outputs from between the cell lines, is that UPN933 appears to have a more noticeable overall response to UPR stress. We speculate that the lower-grade tumor, slightly more akin to normal cells, may have a more dynamic UPR compared to a higher grade tumor that maintains an almost intrinsically elevated stress response [26–28].

A Panther database-assigned list of classifications for particular biological process, molecular function or protein class for the entire dataset is in Supplementary Table S6.

### Ingenuity Pathway Analysis of proteome subsets: canonical pathways

**Figure 5:**
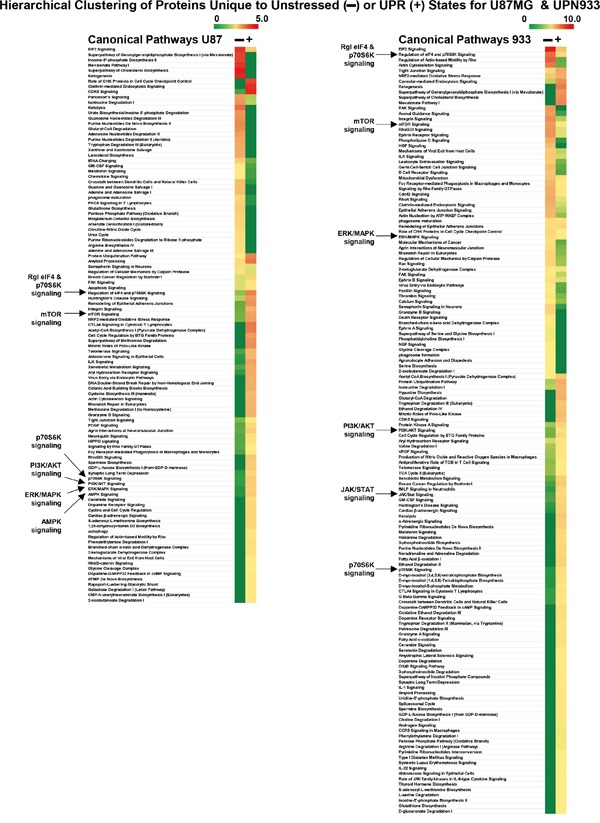
Ingenuity Pathway Analysis “Comparison Analysis” of proteins unique to unstressed versus stressed (UPR-induced) U87MG (left side) and UPN933 (right side) cells (same protein set as in Figure 3) Proteins are grouped by Canonical Pathways prioritized by hierarchical clustering, and scores (−log [p-values]) are shown as heat maps. Signaling pathways further analyzed in Figure 6 are denoted by arrows.

**Figure 6:**
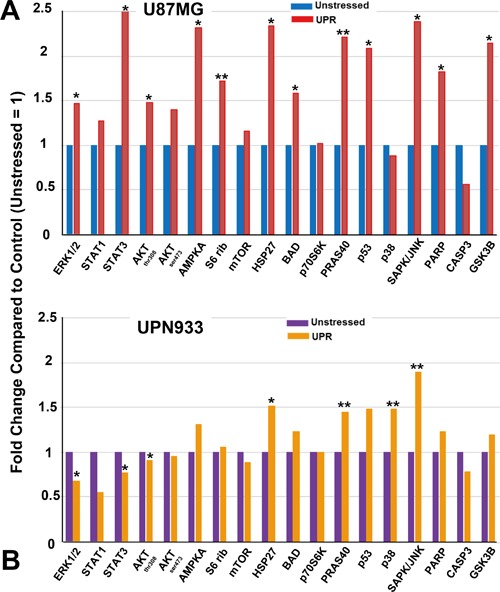
Intracellular signaling array (antibodies vs phospho-proteins and cleaved proteins) probed with unstressed or UPR-stressed U87MG A. or UPN933 B. lysates Luminescent intensities were quantified and presented as fold change compared to control (unstressed lysates set = 1). Standard deviations were less than 25%. Statistics are based on t-tests comparing treated vs controls (* = p<0.05; ** = p<0.01). S6 rib = S6 ribosome.

We employed Ingenuity Pathway Analysis (IPA) to assess potential changes in the top canonical pathways upon stress induction. Proteome subsets inputted were those unique to unstressed and UPR conditions within each cell line (represented in Figure 1B, 1D; 3A), and were analyzed by Comparisons of Core Analyses. Figure 5 shows the Canonical Pathways identified, grouped, and scored by hierarchical clustering. There were 105 and 143 significantly scoring pathways in the U87MG and UPN933 datasets, respectively. Notable pathways in common include predicted stressed reductions in eIF2 signaling (as might be expected with UPR-induced eIF2α phosphorylation), and reductions in semaphorin signaling indicative of de-differentiation [29]. Pathways in common that displayed increased scores in the stressed state include those with few cited connections to the UPR such as CDK5 signaling, tight junction signaling, and telomerase signaling. Pathways known to intersect with the UPR include Huntington's signaling and NRF2 roles in UPR-related oxidative stress, as well as the involvement of ceramide in the UPR in both signaling and membrane function capacities (and as mentioned above, in connection with exosome/extracellular vesicle production [30]). Curiously, glutathione biosynthesis pathway scoring decreases in stressed U87MG while increasing in stressed UPN933. As noted above, this may relate to the more pronounced UPR differential in UPN933 compared to U87MG that may be reflected by redox stress ER glutathione levels [31]. As noted previously, it may also refer to depletion of oxidoreductases during the UPR-induced oxidative stress situation [31]. In Figure 5 we have also highlighted several signaling pathways common to both cell types that receive further analysis in Figure 6.

### Intracellular signaling array analyses

Based on suggestive data from Figure 5, we used unstressed or UPR-induced cell lysates to probe signaling arrays for phosphorylation and cleavage-state changes. Some of these changes reflect findings from Figure 5; for stressed U87MG, there are increases in ERK, STAT, AKT, AMPK, and mTOR phosphorylation. S6 ribosome phosphorylation is also up, indicative of p70S6K activity. Increased phosphorylation is also seen on HSP27, BAD, PRAS40, p53, SAPK/JNK, and GSK3B, and increased PARP cleavage, but reduced CASP3 cleavage. The profile is rather different for UPN933, with comparatively modest phosphorylation increases in AMPK, HSP27, BAD, PRAS40, p53, and GSK3B but a large increase for SAPK/JNK. There is minimal PARP cleavage. This reduced signaling pattern/regulation for ERK, mTOR, and p70S6K follows from IPA data, but runs counter for STAT and AKT signaling. This may be due to increased phosphatases (e.g., PPP2R5E and PGP in stressed UPN933, Supplementary Table S4), differences in process kinetics, and the fact the presence of proteins does not necessarily indicate their activity. Arrays themselves and corroborative Western blots are shown in Supplementary Figure S5. Of note is that pBAD levels in Westerns did not reflect the results seen in the arrays. This may be due to the use of different antibodies for the arrays compared to the Westerns, as well as different antigen conditions (presumably native for the arrays vs reduced/denatured for the Westerns).

## DISCUSSION

The UPR is a cellular mechanism activated under ER stress conditions which drives transcriptional/translational changes in cells. The UPR plays roles in human tumors affecting different aspects of tumorigenesis [32]. However, little is known about the UPR in brain tumor biology [11, 16, 33], particularly the molecular identities involved in downstream processes. We employed mass spectrometry techniques analyzing the global proteomes of two high grade glioma lines under homeostatic and stress conditions. We identified proteomic changes between the two conditions in/within both cell lines, i.e., unstressed versus stressed, and also between two cells lines, i.e., U87MG and UPN933. We observe these changes in all three categories of GO analysis performed (biological process, molecular function, protein class). Overall, we do not find vast changes when assessing the global proteomes. This may be due to a somewhat general phenomenon of tumors as already-stressed entities [11, 16, 34]. Thus, tumor cells may not show dramatic molecular changes during ER stress. Considering the small global changes between the two conditions (~15-25%), we evaluated proteins that are specific for each condition (unstressed vs stressed) and each cell line (U87MG vs UPN933) as better representations of specific functions within each of the conditions/lines. We note that, although the number of proteins within a particular category may vary little from one condition or line to another, the proteins unique to a condition or a cell line may differ as opposed to total number. Therefore, we believe the most powerful utility of our effort is identifying such differences that may point to differences in tumor aggressiveness and effects of the UPR. Nonetheless, if one does consider the “unique” proteins held in common between the cell lines in the unstressed states (Supplementary Figure S6A) and stressed states (Supplementary Figure S6B), the overlaps are relatively small (~2.5% and ~9%, respectively—these proteins are derived from those uniquely identified for each cell line unstressed or stressed, Supplementary Table S3). For the unstressed cells, this essentially verifies that the cell lines are indeed different from each other. However, for the UPR-induced cells, this may seem disconcerting. Still, bioinformatics analysis of those proteins strongly suggests a unified UPR character, particularly in the networks those proteins support (Supplementary Figure S6C), where signaling hubs involving p53, MAPK, ERK/AKT, are prevalent, and reflect both the canonical pathways and signaling molecules identified in this work (Figures 5 and 6).

Evaluation of the data based on total proteins identified, and most all the various categories of the GO parameters, showed more protein identifications for the stressed state in both cell lines. We previously demonstrated that even amidst sustained UPR stress, U87MG quickly overcomes eIF2α phosphorylation, with subsequent protein translation [15]. Curiously, in the various GO analyses performed, the number of phosphatases was increased in stressed UPN933 (Figures 2–4, Supplementary Table 4), including PTPN1, which can mitigate PERK/eIF2α signaling during ER stress [35]. Another protein of interest is the ATPase ASNA1 which facilitates tail-anchored insertion of proteins into the ER membrane (which does not require ongoing protein synthesis)[36].

The number of proteins considered chaperones are higher in stressed cells, as expected [15], validating UPR induction (Figures 2–4, Supplementary Tables S1–S3). A notable difference that may point to an involvement in tumor progression is a decrease in the number of proteins with antioxidant activity in both cell types under stress conditions. Further, stress induction led to loss of identification for all such proteins in U87MG with implications for the tumorigenicity of these cells. This could imply that the cells have sufficient oxidative capacity despite the stress conditions to devote no additional molecular resources to the issue [16]. The antioxidant activities of the proteins identified function in regulation of ROS. It is conceivable that UPR induction with DTT, which generates ROS thru oxidative protein folding [11, 16, 37], may increase utilization of antioxidant proteins, resulting in their net depletion. ROS are known contributors to cancer progression; these data suggest suppression of pro-tumorigenic processes relating to ROS in unstressed cells [38]. Proteins of metabolic processes were also increased under stress; enhanced metabolism was a hallmark of UPR-stressed cells in our previous study [15]. Some proteins link various categories such as ERP44—a chaperone of the protein disulfide isomerase family involved in redox homeostasis with metabolic/catalytic influences [39].

IPA-generated hierarchical clustering of state-specific canonical pathways (Figure 5) revealed both similarities and differences between cell line responses, several of which we noted above. Interestingly, dichotomous pathways include RhoGDI signaling and signaling by Rho family GTPases (up in stressed U87MG, down in stressed UPN933), but Rho-related signaling accounts are rare regarding the UPR [40–43]. As mentioned, there are numerous pathways that increase scores in the stressed state, but have few cited relationships to the UPR such as CDK5 signaling (with MEKK1 activation leading to JNK-driven apoptosis [44]), tight junction signaling (vague in terms of the UPR, but with involvement in neurodegenerative states [45]), and telomerase signaling and UPR [46]. Better known UPR connections to increased signaling pathway scores include Huntington's signaling [47, 48], NRF2 in oxidative stress [49, 50], and roles played by ceramide in the UPR [51, 52].

IPA identified major signaling pathways that were further tested with antibody arrays (Figure 6). Consistent with GO analyses and IPA, the profiles differ between the two cell lines. In pathways with substantial overlap between the two lines, the literature remains confusing. The AMPK pathway, a known metabolic sensor with conflicting contributions in cancer [13], engages in cross-talk with the UPR with differing impacts ranging from attenuating UPR outcomes [53, 54], to overcoming UPR effects [55], to contextually differential effects [56], to cooperative effects [57–59]. HSP27 is phosphorylated during ER stress [60], and both HSP27 and S6 ribosome are phosphorylated during the stress of gravitational unloading [61], but little else is known. PRAS40/AKT1S1 is a target of AKT signaling that may de-repress mTOR signaling (while mTOR complexes also promote AKT signaling [62]), although those interactions and outcomes are complicated [63]. Indeed, muscle-cell ER stress reduced PRAS40 phosphorylation in an anabolic resistance model [64]; our previous work [15], however, showed increased amino acid uptake for UPR-induced U87MG. p53 also has complicated stress relationships, where the UPR can promote p53 accumulation, but with cell-cycle arrest [65]. Chemotherapy-driven UPR resulted in a similar molecular phenotype to those presented here, including JNK phosphorylation and accumulation of p53, but with cytotoxicity [66]. Others have shown p53 destabilization (with different phosphorylation events) following ER stress [67].

Apoptosis always looms as an UPR outcome, but tumors confound these events with altered regulations of sometimes conflicting pathways [26]. Our work also yields potentially surprising results, particularly in areas where little previous research exists. HSP27 is downstream of p38/MAPK signaling [68], and both p38 and JNK are implicated in ER stress signaling [69]. JNK connections to BAD phosphorylation (which is anti-apoptotic on Ser112) may involve the latter's sequestration by 14-3-3 proteins to determine survival outcomes [70] (we identified numerous 14-3-3 family members here, Supplementary Tables S1, S2). Previous reports suggest that ER stress levels (DTT-induced) may lead to prolonged JNK activation with apoptotic outcomes [71]. UPR induction in our tumor cells indeed led to prolonged JNK phosphorylation, but without ensuing cell death [15] (and data not shown). PARP is a DNA-repair enzyme whose cleavage indicates apoptosis, particularly during stress [72]. It is a target of CASP3, but also CASP7, cathepsins [73], and calpains [74], the latter two families identified in proteomics (Supplementary Tables S1, S2). Notably, CASP3 cleavage/activation is reduced in our stressed samples, suggesting alternate routes. Despite increased outputs of apoptosis-related proteins in stressed states (Figures 3–5), the differential effects of UPR induction on the cells ultimately did not lead to cell death. GSK3B phosphorylation is functionally inhibitory and anti-apoptotic, improving survival of UPR-stressed tumor cells (uniquely stressed by activated A2M [75]). In other scenarios, however, UPR-induced cells activate (dephosphorylate) GSK3B [76, 77]. PKB/AKT is one of the major hubs in signal transduction with impacts in tumorigenesis and metabolism [78]. In terms of U87MG, prior work in other cells predicts that the UPR activates ERK and AKT, promoting survival pathways [79], although this is neither universal nor straightforward [80], particularly in toxic treatment settings [81, 82]. Curiously, PI3K and phospho-AKT (Thr 308) have been implicated as distinguishing GBMs from lower grade gliomas, and phospho-BAD may be considered important in resistance to apoptosis [83].

STAT3 is a transcription factor frequently activated in cancer cells via cytokine and growth factor signaling, with resultant tumor gene expression for survival, angiogenesis, and invasive/metastatic phenotypes [84]. IRE1 binding leads to phosphorylation of STAT3 in hepatocytes [85], but in murine astroglia, ER stress reduced phospho-STAT3 [86]. On the other hand, ER-stressed pancreatic cells expressed transcription factor AATF, which upregulated AKT expression via STAT3, preventing cell death [87]. Evidently, there are multiple pro- and anti-apoptotic inputs into these UPR scenarios that nonetheless in these glioma cells result in cell survival; these remain areas of further study.

Pharmacologic targeting of the UPR is a burgeoning area of research with potential therapeutic interventions [88, 89]. As we have noted before, bortezomib, an FDA-approved proteasome inhibitor, was used in clinical trials for gliomas (alone and in combination) with unsatisfying results [11] (see also [90]). Poor blood-brain barrier penetrance has been suggested as reason for these disappointing outcomes [91]. Bortezomib is a known inducer of the UPR, possibly by repressing endoplasmic reticulum associated degradation (ERAD)[92]. Bortezomib and other proteasome inhibitors have additional effects on glioma cells that may involve UPR activation, including upregulated VEGF production in “stem-like cells”[93], and p38 MAPK activation [94]. While the p38 status varied between cell lines in our study, the concept of over-stressing cells to the point of apoptosis is appealing, but it is unclear to what extent tumor cells can tolerate such stresses, particularly *in vivo*. For instance, PI3K/AKT activation was considered a major mechanism for failure of combined bortezomib/ABT-737 to drive apoptosis in glioma cells, particularly those with PTEN mutations (eg, U87MG) [95]. Our results implicate activation of AKT and downstream players following UPR induction in U87 cells, thus suggesting that targeting of those pathways may be important in reducing the chemo-resistance impact of the UPR [15]. Furthermore, we found a number of histones and histone deacetylases in our proteomic efforts (Supplementary Tables S1 and S2), with studies suggesting that bortezomib combined with HDAC inhibitors may show efficacy against glioma cells [96]. In general, targeting the UPR for anticancer effects appears promising, but our work suggests that caveats may apply in terms of further promoting even more activated and resistant stress responses.

Analyses of proteins grouped within the GO categories, IPA, and signaling pathways evaluated here show specificity for cell line and/or condition for some processes/functions while remaining redundant for others. The specificity of signaling pathways and processes is clearly dictated by the cell line and condition. It is unclear how the changes in the stressed cells are pro- or anti-tumorigenic based on this study, and the literature reveals similar disparities. The work warrants *in vitro* and *in vivo* follow-up experiments assessing the UPR effects.

Our work establishes a strong foundation for future studies further exploring the differences observed here, enhancing our understanding of UPR effects on brain tumor biology, and effects on different types of brain tumors. Identity validation, relative quantification, and functional cell-based approaches are necessary for a clearer and more conclusive understanding of cellular stressor effects and ensuing signaling pathway engagement in glioma biology that would ultimately lead to better therapies.

## MATERIALS AND METHODS

### Cell culture

U87MG is from ATCC (Manassas, VA). UPN933 cells were cultured from an anaplastic oligodendroglioma (WHO grade III) obtained on a study approved by the Colorado Combined Institutional Review Board (COMIRB), #95-100. Cells were cultured under “stem-cell conditions” as described [15, 97] and in Supplementary Materials. STR analysis and verification were performed in Nov 2014 by the UCCC PPSR.

### Induction of the unfolded protein response (UPR)

We induced the UPR with 1mM dithiothreitol (DTT), 4 hrs, as described [15]. After treatment, cells were washed and incubated for 24 hrs in DTT-free media. Cells were harvested by centrifugation (1100 x g, 5 min); supernatant was aspirated and cell pellets rinsed twice in PBS.

### SDS-PAGE

Cell pellets were lysed in 2 mL RIPA buffer (Sigma-Aldrich, St Louis, MO) containing phosphatase and protease inhibitors (Roche, Indianapolis, IN). Cell lysate was centrifuged (12,000 x g, 10 min, 4°C); supernatant was collected and stored at −80°C until used. We performed SDS-PAGE on equal quantities of RIPA lysate (BioRad, Hercules, CA); gels were stained with Coomassie blue dye. Replicate samples were used for each condition in each cell line analyzed.

### Western blotting

Western blots of lysates were run as described [15] (also, Supplementary Materials).

### Mass spectrometry/proteomics

Coomassie-stained gel bands were cut out and de-stained using 50mM ammonium bicarbonate/50% acetonitrile (50mM ABC/50% ACN, Sigma-Aldrich) solution, then rinsed in water. Bands were dehydrated using 100% ACN, then reduced for 45 min, 60°C with 10mM DTT in 50mM ABC. Bands were alkylated using 50mM iodoacetamide in 50mM ABC (Sigma-Aldrich) (25 min, RT, in darkness); bands were washed with ABC (15 min, RT). Bands were digested with 0.3μg of trypsin in 50mM ABC (12 hr, 37°C). Peptide extraction, separation, and MS analysis were previously described [98] (also, Supplementary Materials). Venn diagrams were generated with Venny 2.1.0 (http://bioinfogp.cnb.csic.es/tools/venny/).

### Panther Database

Panther Database version 9.0 (http://www.pantherdb.org/panther/ontologies.jsp) was used to generate gene ontology profiles of identified proteins based on biological process, molecular function and protein class for each condition and each cell line.

### Ingenuity Pathway Analysis

Ingenuity Pathway Analysis (IPA) (http://www.ingenuity.com/) Core Analysis was used for evaluation of protein datasets. IPA Comparison Analysis compared similarities and differences between the proteins unique to unstressed/stressed conditions within each cell line. Data are presented as hierarchical heat maps.

### Intracellular signaling arrays

Cells were left unstressed or were UPR-stressed (1 mM DTT, 4 hrs); 24 hrs later, lysates were prepared and incubated on PathScan Intracellular Signaling Arrays (Cell Signaling Technologies, Danver MA, USA) according to manufacturer's directions. Arrays were scanned and quantified using a FluorChem Q Imager III device (ProteinSimple, Santa Clara CA).

## SUPPLEMENTARY FIGURES AND TABLES














