# Supplementary material for: Proteomic analyses of brain tumor cell lines amidst the unfolded protein response
Source: Oncotarget. 2016 Jun 14;7(30):47831–47. doi: 10.18632/oncotarget.10032 (PMC5216982; doi:10.18632/oncotarget.10032)
Supplement: Supplementary file 1 [file oncotarget-07-47831-s001.pdf]

Proteomic analyses of brain tumor cell lines amidst the unfolded protein response

SUPPLEMENTARY DATA

U87MG and UPN933 cells were cultured in Neurobasal A medium without Phenol Red (Invitrogen Technologies, Grand Island, NY) and with addition of the following supplements: B-27 Supplement, N-2 Supplement, 0.5 ng/ml recombinant Epidermal Growth Factor (all from Invitrogen Life Technologies) and 0.5 ng/ml bovine Fibroblast Growth Factor (Thermo Fisher Scientific, Waltham, MA). Cells were cultured at 37°C and 5% CO_2 (Sigma Aldrich, St. Louis, MO). Cells were incubated in DTT-containing media for 4 hours at 37°C and 5% CO_2. Following the 4-hour incubation, cells were rinsed 2 times with 1X phosphate buffered saline (PBS, Gibco Life Technologies, Grand Island, NY) then incubated for 24 hours in DTT-free media. The cells were collected and centrifuged at 1100 x g for 5 minutes, supernatant was aspirated and the cell pellet rinsed 2 times with 1X PBS.

Following SDS-PAGE, proteins were transferred to nitrocellulose membranes (iBlot system, Invitrogen). Membranes were blocked by incubation in 5% milk/PBS+ 0.5 % Tween-20 (PBST) overnight at 4°C. The blots were then probed with the following antibodies (from Enzo/ Assay Designs, Farmingdale, NY): anti-GRP94 (SPA-850), anti-calnexin (SPA-860), anti-GRP78 (SPA-826), anti-ERp72 (SPA-720), anti-calreticulin (SPA-601), anti-GRP94 (SPA-850); 1:1000 dilutions in 1% milk/ PBST. From Abcam (Cambridge, MA): anti-CHOP/ GADD153 (ab53081), 1:1000 dilution. From Immuno-Biological Laboratories (Gunma, Japan): anti-ORP150/ GRP170, 1:1000 dilution. From Biomol International (Plymouth Meeting, PA), anti-HERPUD, 1:2000 dilution. From Santa Cruz Biotechnology (Santa Cruz, CA): anti-XBP-1 (sc7160), 1:1000 dilution. From Abnova (Taipei, Taiwan): anti-ATF6 (MAB0082,) 1:1000 dilution. From Cell Signaling Technologies (Danver MA, USA): anti-pan AKT(Thr308) (4691); anti-pAKT (13038); anti-AMPKA (D63G4); anti-pAMPKA(Thr172) (40H9); anti-BAD (D24A9); anti-pBAD(Ser112) (40A9); anti-pGSK3B(Ser9) (D85E12); anti-SAPK/JNK (9252); anti-pSAPK/JNK(Thr183/Tyr185) (9251); anti-STAT3 (12640); anti-pSTAT3(Tyr705) (D3A7). All are rabbit antibodies, used at 1:500 dilution. From Sigma Aldrich: anti-β-actin antibody (A5316) was used as a loading control, 1:1000. Appropriate species-specific secondary antibodies (anti-rat, anti-mouse, or anti-rabbit IgG-HRP conjugates) were used at 1:5000 dilutions (all from GE HealthSciences/Amersham, Piscataway, NJ, USA). Following secondary antibody incubations, membranes were washed in PBST, dH2O, and incubated in enhanced chemiluminescent substrate (Pierce Chemical Co., Rockford, IL) prior to electronic imaging in a FluorChem Q Imager III device (ProteinSimple, Santa Clara CA). Densitometry measurements for Western blots were made with ImageJ (https://imagej.nih.gov/ij/).

Trypsin digest solution was collected in a clean centrifuge tube the following morning. Five percent formic acid in ACN (5% FA/100% CAN, Sigma-Aldrich) was used to extract the peptides for 20 minutes in a sonicating water bath. The solution was collected, combined with the trypsin digest and extraction repeated once more. Once again, the extraction solution was added to the trypsin digest and the solution was dried to completion using a speed vacuum. The pellet was resuspended in 0.1% 0.1 % FA.

Five µl of the trypsin digests were separated using 5–50% ACN gradient over 120 min on a C18 column, 15x0.1mm, at a flow rate of 500nl/min (Michrom/ Bruker, Auburn, CA). MS/MS spectra were collected using the Amazon Speed ion ETD trap equipped with CaptiveSpray nanoBooster ionization source (Bruker Daltonics, Freemont, CA) housed at the University of Colorado School of Pharmacy Mass Spectrometry Core Facility. Acetonitrile enriched nitrogen gas was used as a sheath gas to increase the charge state of peptide ions and enhance identifications. Data processing was performed using ProteinScape 3.1. Database searches were performed against all of the human entries in the SwissProt database (Swiss Prot_2014.06) using the Mascot Server (v2.4.1) using 0.6 Da peptide mass tolerance and 0.5 Da MS/MS tolerance allowing for 1 missed cleavage and modifications for oxidation of methionine and carbamidomethylation of cysteine. Protein identification was considered significant if at least 2 unique peptides were used for identification.

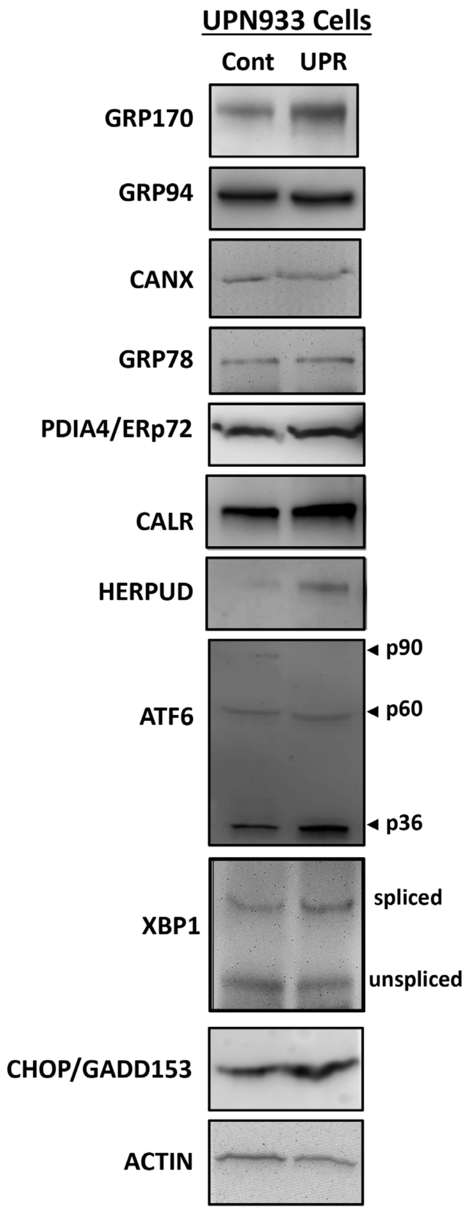

Supplementary Figure S1: Upregulation of UPR-regulated proteins in UPN933 cells. Primary anaplastic oligodendroglioma cells were grown under serum-free conditions and were treated (or not, "Cont") with 1 mM DTT ("UPR") for 4 hrs. Cell cultures were harvested, and cells lysed as described. Proteins were separated on SDS-PAGE and Western blotted and probed with the antibodies listed. Upregulation of some of the UPR components is evident. Actin probe is used as a loading control. Images have been "inverted" (converting from light bands/spots on a dark background as seen with ECL reagents to dark bands/spots on a light background as typically done from Western blot viewing) and adjusted with brightness/contrast for improved viewing. Notably, brightness/contrast adjustments were not extensive, as the images do not appear pixelated. Images may have been slightly rotated for square corners. Images have been cropped (without loss of information) to reduce the overall size of the figure. Images have had box outlines added to improve aesthetics.

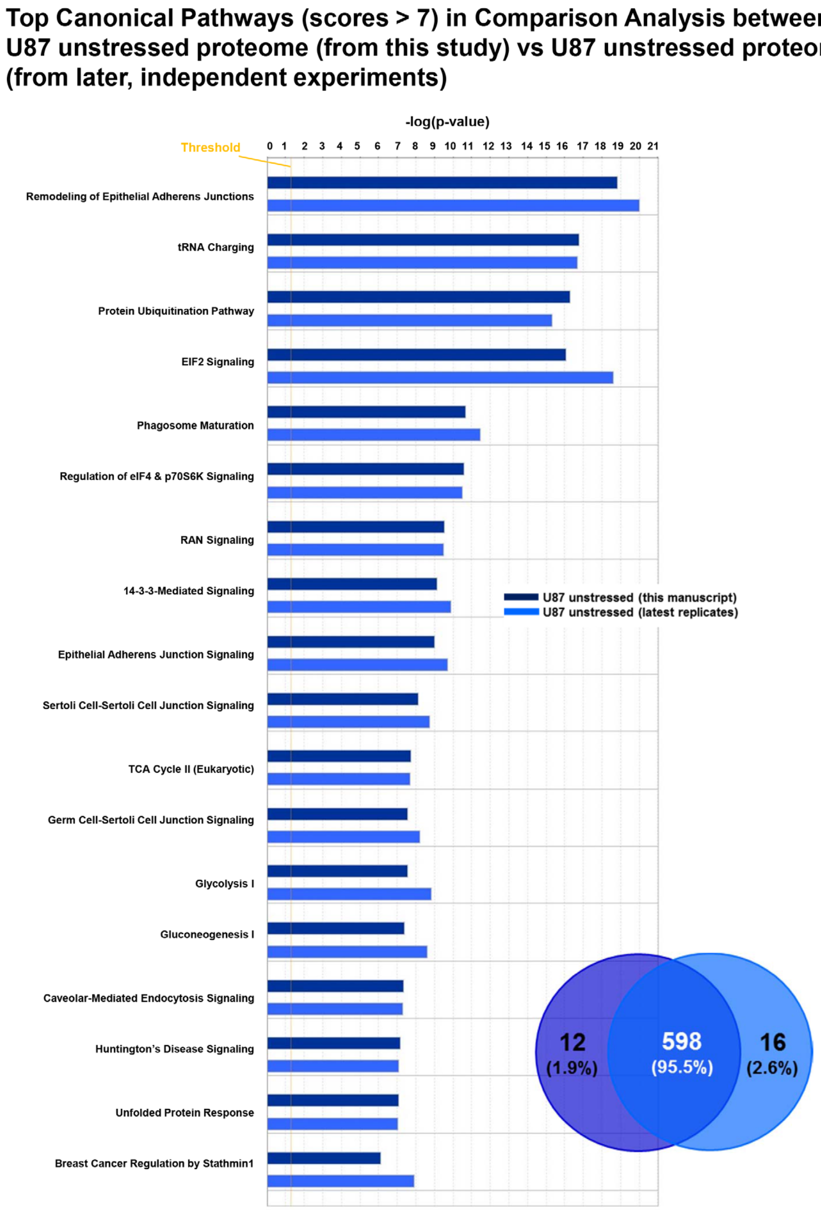

Supplementary Figure S2: The total proteomes of U87MG cells in their unstressed states from independent replicates (those used in this study, and those performed separately) were utilized in IPA's Comparison Analysis. Scores (-log[p-value] from Fisher's exact test) are graphed; for brevity, only those categories >7 are shown ("Threshold" is set as the limit of significance, at 1.25; 123 significantly scoring categories were determined). Venn diagram shows overlaps and unique numbers of proteins identified between the sets of replicates.

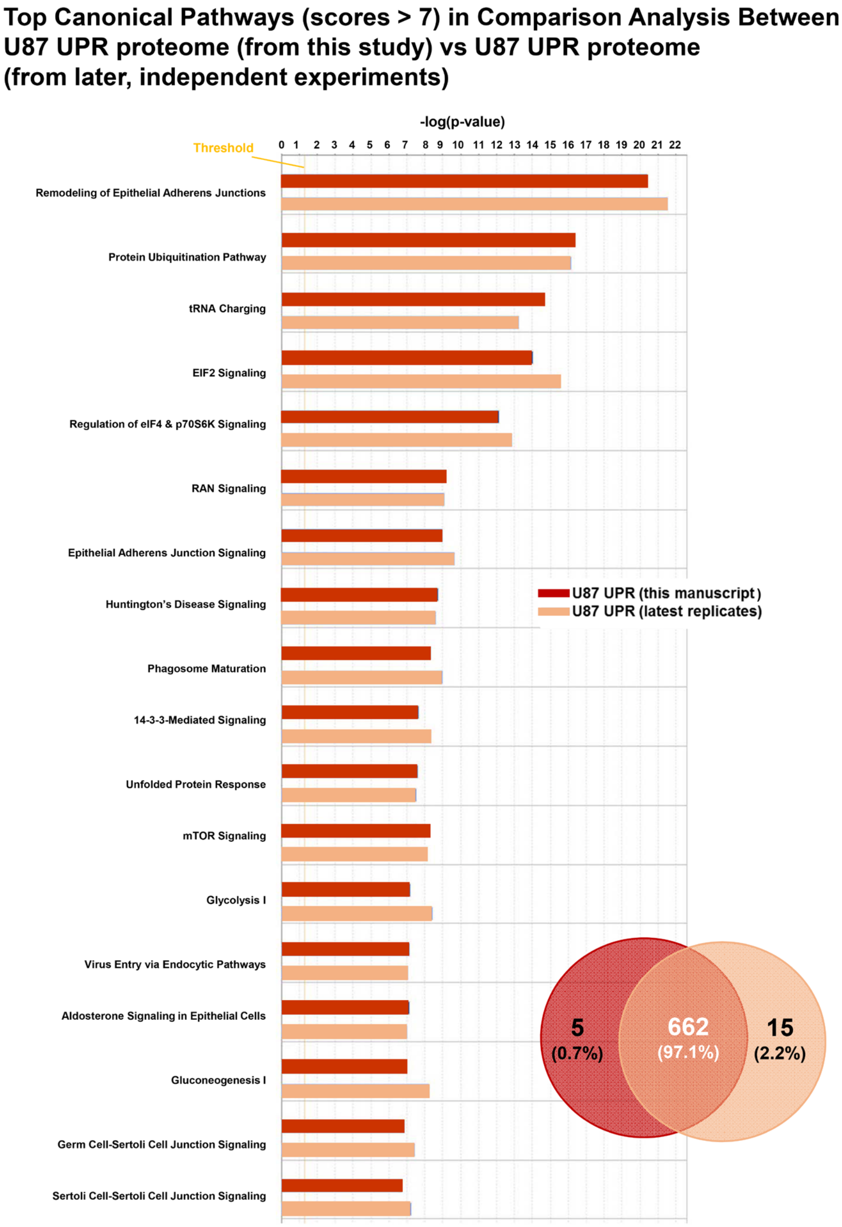

Supplementary Figure S3: The total proteomes of U87MG cells in their UPR-induced states from independent replicates (those used in this study, and those performed separately) were utilized in IPA's Comparison Analysis. Scores (-log[p-value] from Fisher's exact test) are graphed; for brevity, only those categories >7 are shown ("Threshold" is set as the limit of significance, at 1.25; 142 significantly scoring categories were determined). Venn diagram shows overlaps and unique numbers of proteins identified between the sets of replicates.

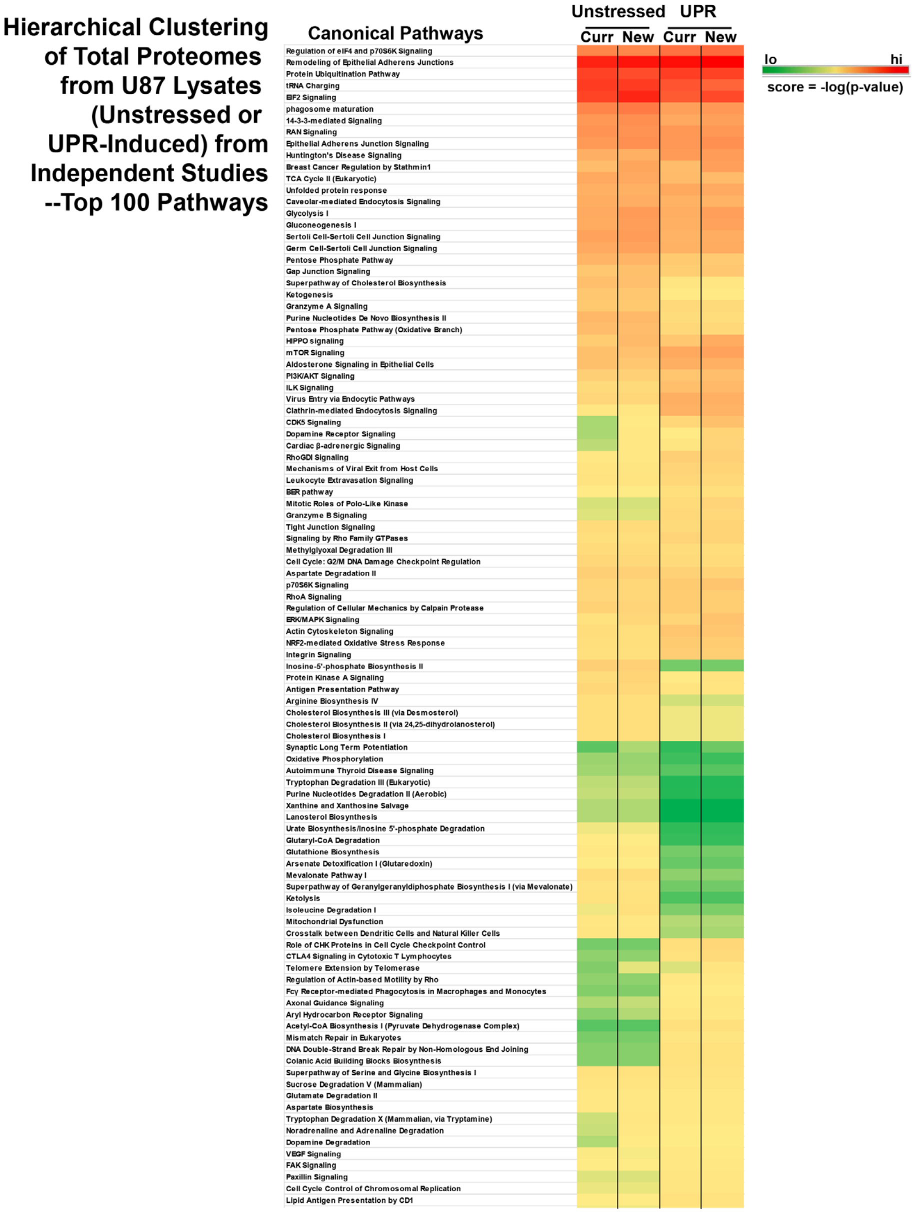

Supplementary Figure S4: Heat map presentation of IPA Comparison Analyses via hierarchical clustering of the top 100 Canonical Pathways identified from the two replicate sets of proteomic data for the current study ("Curr") and the two replicate sets from another ("New") study. Data from the total proteins identified from unstressed and stressed conditions are shown.

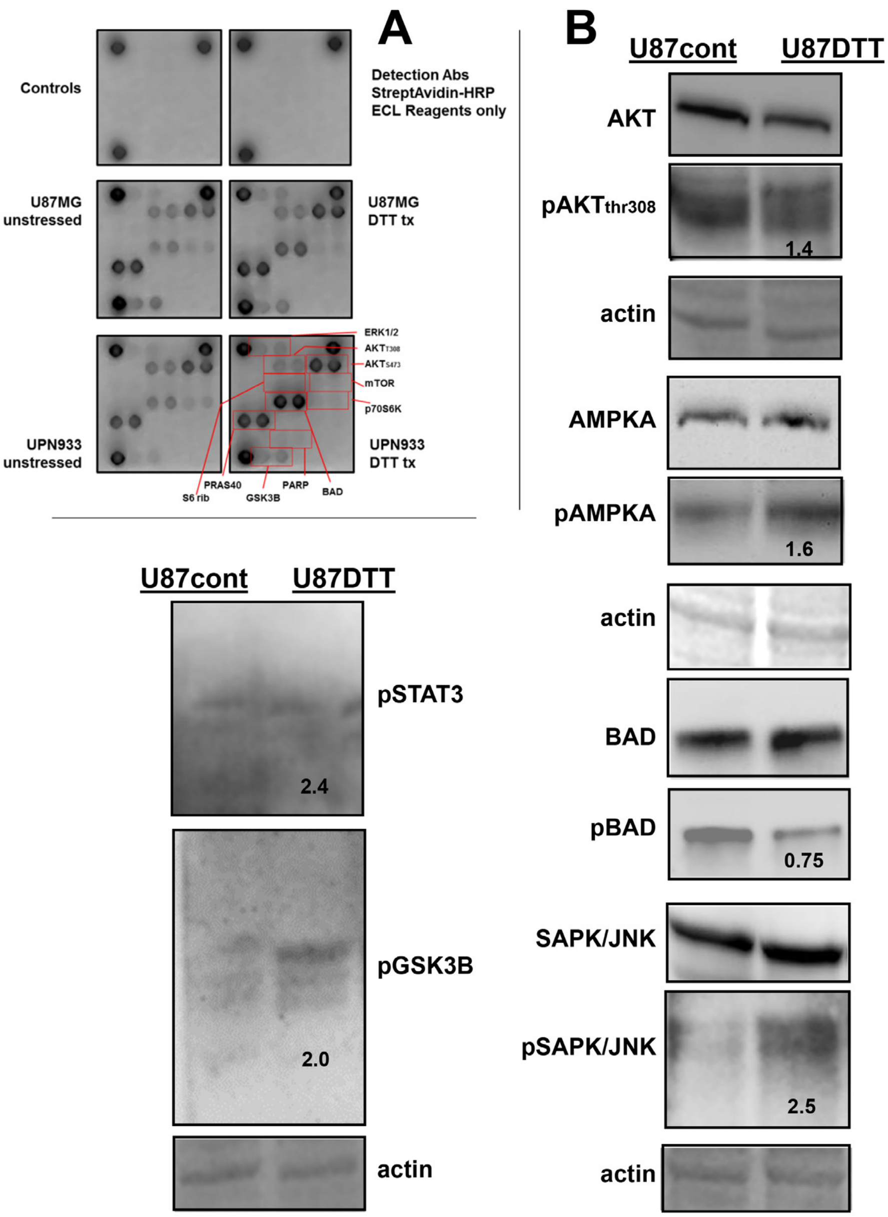

Supplementary Figure S5: Intracellular signaling array data and correlative Western blots. A. Intracellular signaling arrays with control incubations (upper two panels), incubated with U87MG lysates from untreated cells (middle left) or cells treated with DTT (middle right), or incubated with UPN933 lysates from untreated cells (bottom left) or cells treated with DTT (bottom right). Bottom right block indicates some of the relevant proteins notable in the array. **B.** Western blots of control (untreated) U87MG cell lysates and DTT-treated cell lysates probed for various proteins and their phosphorylated constituents (along with actin loading controls). Numbers in the DTT-treated lanes indicate the fold change of phospho-protein levels measured by ImageJ quantitation versus the relative (total) protein quantities (relative to actin) in the untreated state. Images have been "inverted" (converting from light bands/spots on a dark background as seen with ECL reagents to dark bands/spots on a light background as typically done from Western blot viewing) and adjusted with brightness/contrast for improved viewing. Notably, brightness/contrast adjustments were not extensive, as the images do not appear pixelated. Images may have been slightly rotated for square corners. Images have been cropped (without loss of information) to reduce the overall size of the figure. Images have had box outlines added to improve aesthetics.

A

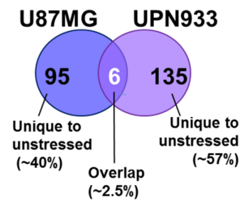

The differences in proteins unique to the un-stressed state between U87 and UPN933 are evident in this Venn diagram; it essentially indicates that the cell lines are indeed different. Ingenuity Pathway Analysis Core Analysis (data not shown)of the 6 proteins in the overlap showed only 6 Canonical Pathways that were barely above threshold, and only 2 networks with very limited numbers of proteins (and again, low scores).

B

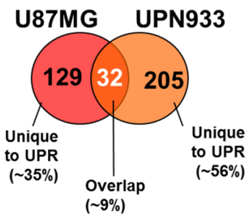

A seemingly small number of proteins (~9%) overlap in the UPR states from both cell lines. However, IPA Core Analysis of the 32 proteins reveals that the top five Canonical Pathways (all with scores >3x over threshold) are found in the Canonical Pathways of either/both U87MG and UPN933 stressed states (Figure 5), suggesting important roles in the UPR.

C

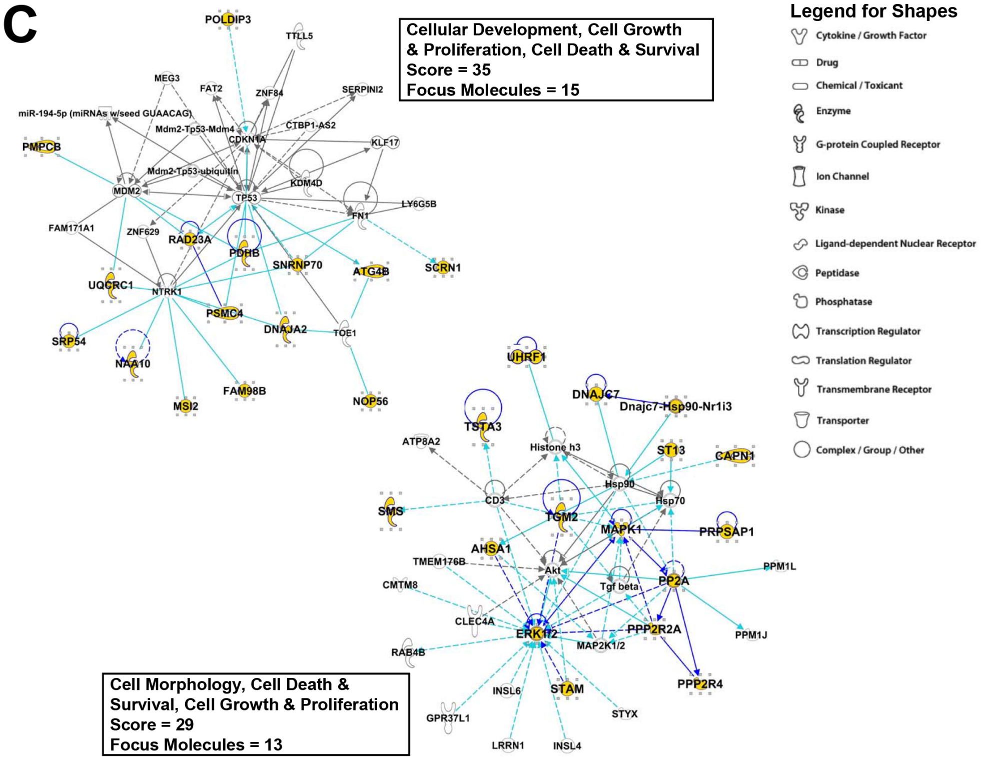

Supplementary Figure S6: Comparisons of the shared proteins from those unique to U87MG and UPN933 unstressed/stressed states (derived from Supplementary Table S3). A. Proteins that are unique to unstressed U87 cells (compared to stressed U87 cells) are merged in a Venn diagram with proteins that are unique to unstressed UPN933 cells (compared to stressed UPN933 cells). The proteins from the overlap were analyzed by Core Analysis using Ingenuity Pathway Analysis algorithms. Due to relatively low protein numbers and lack of relationships, little information is discernable. **B.** Proteins that are unique to UPR-stressed U87 cells (compared to unstressed U87 cells) are merged in a Venn diagram with proteins that are unique to stressed UPN933 cells (compared to unstressed UPN933 cells). The proteins from the overlap were analyzed as in A). **C.** Proteins clustered within the top 2 Networks/Associated Functions as derived from IPA algorithms from the overlap proteins in B) are shown as members of "interactomes". Proteins identified in this study are labeled in larger bold font and associated gold fill. Direct connections are shown in solid lines; indirect interactions are shown as dashed lines ("edges"). Connections between proteins identified in this proteomic screen are shown in dark blue; interactions between proteins we identified and other proteins in the network are in turquoise. Protein shapes = functional categories shown in the legend at the right. Network scores (Fisher's exact test, -log [p values] shown) and number of focus molecules (acting as focal point "seeds") are indicated.

Supplementary Table S1: All protein identifications for U87MG cell replicate samples under homeostatic and stressed (DTT Tx) conditions. Proteins from 2 independent replicate lysates of cells either left unstressed or UPR-induced with DTT were identified by mass spectrometry techniques. "ID"/Accession numbers are from the UniProt Knowledge Base (Swiss-Prot/TrEMBL, http://us.expasy.org/sprot/). MW = protein predicted molecular weight (by amino acid) in kilodaltons; pI = predicted isoelectric point; score = protein score in Mascot format; #peptides = number of peptides identified; SC[%] = sequence coverage, percentage ; RMS90[ppm] = root mean square 90% confidence value.

See Supplementary File 1

Supplementary Table S2: All protein identifications for UPN933 cell replicate samples under homeostatic and stressed (DTT Tx) conditions. Proteins from 2 independent replicate lysates of cells either left unstressed or UPR-induced with DTT were identified by mass spectrometry techniques. "ID"/Accession numbers are from the UniProt Knowledge Base (Swiss-Prot/TrEMBL, http://us.expasy.org/sprot/). MW = protein predicted molecular weight (by amino acid) in kilodaltons; pI = predicted isoelectric point; score = protein score in Mascot format; #peptides = number of peptides identified; SC[%] = sequence coverage, percentage ; RMS90[ppm] = root mean square 90% confidence value.

See Supplementary File 2

Supplementary Table S3: Proteins unique to unstressed or stressed condition for each cell line. Proteins identified that are unique to U87MG cells unstressed vs U87MG cells stressed (UPR-induced) are listed. Proteins identified that are unique to UPN933 cells unstressed vs UPN933 cells stressed (UPR-induced) are also listed. Proteins are posted by accession number and protein name.

See Supplementary File 3

Supplementary Table S4: Proteins unique to unstressed or stressed condition for each cell line, listed by GO categories in Biologic Process, Molecular Function, or Protein Class. Proteins from unstressed vs stressed U87MG cells were compared and categorized. Similarly, proteins from unstressed vs stressed UPN933 cells were compared and categorized. Proteins are posted by accession number and protein name.

See Supplementary File 4

Supplementary Table S5: Proteins unique in comparing each cell line for unstressed and stressed conditions. Proteins identified that are unique to U87MG cells unstressed vs UPN933 cells unstressed are listed. Proteins identified that are unique to U87MG cells stressed (UPR-induced) vs UPN933 cells stressed (UPR-induced) are also listed. Proteins are posted by accession number and protein name.

See Supplementary File 5

Supplementary Table S6: Proteins unique comparing each cell line for unstressed and stressed conditions, listed by GO categories in Biologic Process, Molecular Function, or Protein Class. Proteins from unstressed U87MG vs unstressed UPN933 cells were compared and categorized. Similarly, proteins from stressed (UPR-induced) U87MG cells vs stressed UPN933 cells were compared and categorized. Proteins are posted by accession number and protein name.

See Supplementary File 6
